# The Conditional Nature of Genetic Interactions: The Consequences of Wild-Type Backgrounds on Mutational Interactions in a Genome-Wide Modifier Screen

**DOI:** 10.1371/journal.pgen.1003661

**Published:** 2013-08-01

**Authors:** Sudarshan Chari, Ian Dworkin

**Affiliations:** 1 Program in Ecology, Evolutionary Biology and Behavior, Michigan State University, East Lansing, Michigan, United States of America; 2 Department of Zoology, Michigan State University, East Lansing, Michigan, United States of America; 3 Program in Genetics, Michigan State University, East Lansing, Michigan, United States of America; University of Michigan, United States of America

## Abstract

The phenotypic outcome of a mutation cannot be simply mapped onto the underlying DNA variant. Instead, the phenotype is a function of the allele, the genetic background in which it occurs and the environment where the mutational effects are expressed. While the influence of genetic background on the expressivity of individual mutations is recognized, its consequences on the interactions between genes, or the genetic network they form, is largely unknown. The description of genetic networks is essential for much of biology; yet if, and how, the topologies of such networks are influenced by background is unknown. Furthermore, a comprehensive examination of the background dependent nature of genetic interactions may lead to identification of novel modifiers of biological processes. Previous work in *Drosophila melanogaster* demonstrated that wild-type genetic background influences the effects of an allele of *scalloped (sd)*, with respect to both its principal consequence on wing development and its interactions with a mutation in *optomotor blind*. In this study we address whether the background dependence of mutational interactions is a general property of genetic systems by performing a genome wide dominant modifier screen of the *sd^E3^* allele in two wild-type genetic backgrounds using molecularly defined deletions. We demonstrate that ∼74% of all modifiers of the *sd^E3^* phenotype are background-dependent due in part to differential sensitivity to genetic perturbation. These background dependent interactions include some with qualitative differences in the phenotypic outcome, as well as instances of sign epistasis. This suggests that genetic interactions are often contingent on genetic background, with flexibility in genetic networks due to segregating variation in populations. Such background dependent effects can substantially alter conclusions about how genes influence biological processes, the potential for genetic screens in alternative wild-type backgrounds identifying new loci that contribute to trait expression, and the inferences of the topology of genetic networks.

## Author Summary

Examining the consequences of how one mutation behaves when in the presence of a second mutation forms the basis of our understanding of genetic interactions, and is part of the fundamental toolbox of genetic analysis. Yet the logical interpretation of such mutational interactions depends on the generality of such findings. A small number of studies have demonstrated that factors such as the wild-type background in which the mutations are studied can have a profound impact on the observed phenotype of both specific effects of the mutation and the interactions between mutations. However, whether such findings are a common property of genetic interactions was unknown. We tested the generality of the background dependence of interactions between mutations and observed that the vast majority of the interactions were highly dependent on the wild-type background in which they are observed. We demonstrate that the same regions of the genome that contribute to the differences observed in the degree of severity of the mutational effect appear to also be responsible for the background dependence of the interaction.

## Introduction

Fundamental to the logic of genetic analysis is that the observed variation in a phenotype for a genetically mediated trait is causally linked to one or more DNA lesions/variants. However, it is well known that the phenotypic effects of many individual mutant alleles are context dependent, with respect to environmental influences, as well as the “wild-type” genetic background in which the mutation is observed. Indeed, genetic background has long been known to influence observed phenotypic expression across traits, organisms, and a range of allelic effects, including hypomorphs, amorphs/nulls and neomorphs [1]–[9]. These results make it clear that the phenotypic effects of a mutation (i.e. penetrance and expressivity) are themselves “complex traits”, subject to environmental and polygenic influences [1]. Far beyond being a minor curiosity in genetics, the background dependent effects of a number of mutations have been at the heart of debates over the conclusions and the ability to replicate key findings from several studies, including the genetics of life span [10]–[14], stress tolerance [15]–[17] and pigmentation [18]–[20].

**Figure 1:**
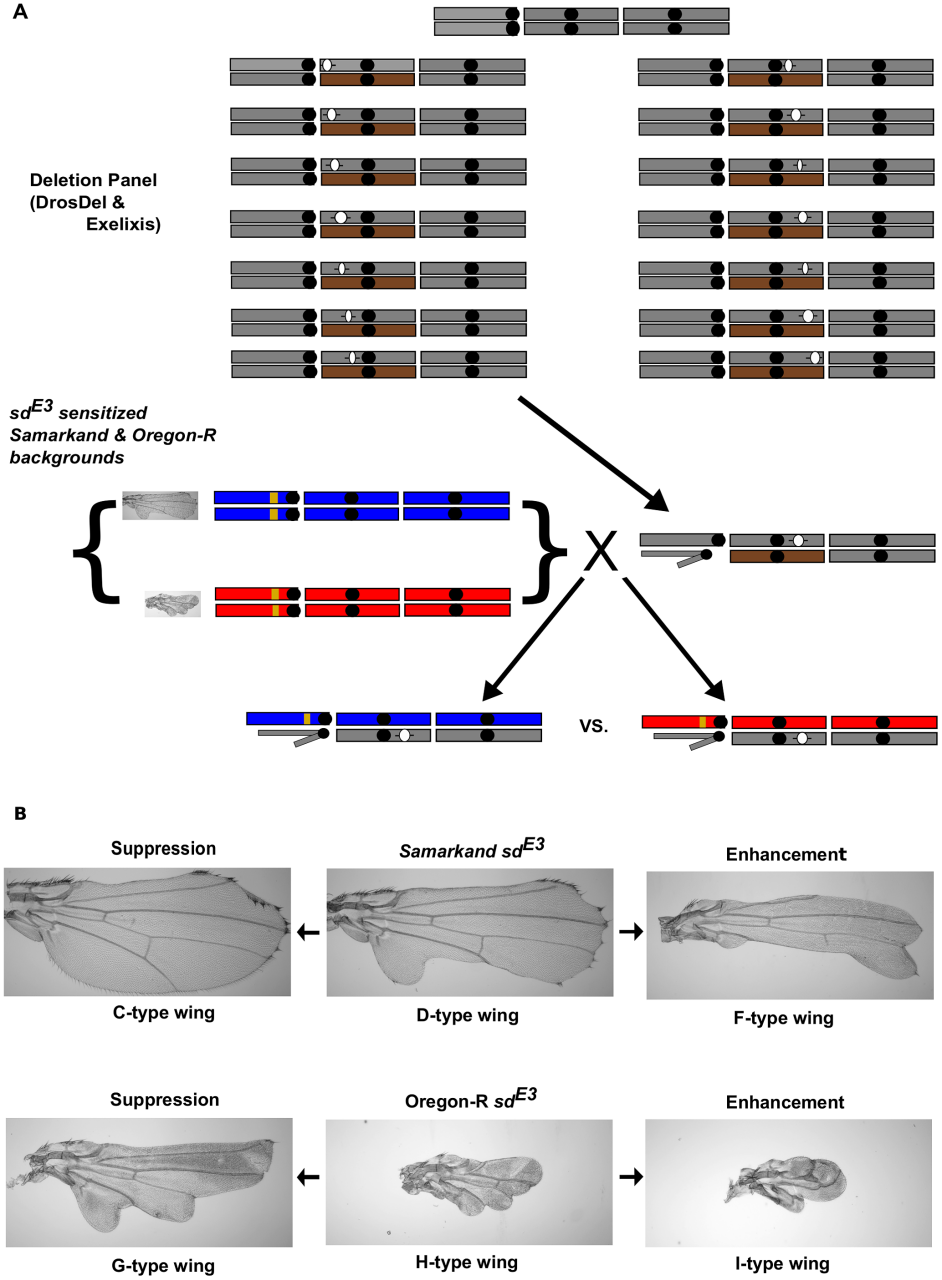
Genetic background effects influence *sd^E3^*, and are used for a dominant modifier screen. A) Outline of the modifier screen employed in this study (illustrated for 2^nd^ chromosome deletions). Using the DrosDel and Exelixis Deletion collections, male deletion–bearing (denoted with -()- ) flies were crossed to females homozygous for the *sd^E3^* mutation from each wild-type genetic background, Samarkand (blue) and Oregon-R (red). Male offspring that were hemizygous for the *sd^E3^* allele and heterozygous at all other loci, including the deletion, were compared between the two genetic backgrounds. Thus we were scoring male flies hemizygous for *sd^E3^*, and heterozygous for the deletion. The co-isogenic progenitor wild-type strains was used for control crosses. Each grey rectangle represents a chromosome (X, 2 & 3 from left to right), with centromeres (black dots), and balancer chromosomes (brown rectangles). Yellow represents the *sd^E3^* mutation and closely linked genomic region on the X chromosome. B) The effect of genetic background on the phenotypic expression of the *sd^E3^* allele, and examples of suppression and enhancement of this allele in each background. Letters beside each image represent the semi-quantitative scores assigned to wings (all figures taken at 40× magnification).

Although the basic influence of genetic background on the expressivity of mutations is well documented, the wider consequences of such influences are poorly understood [21]. In particular, the extent to which wild-type background influences the magnitude and sign of genetic interactions remains unclear. Research to date addressing this question [4], [22], [23], has largely focused on a small set of mutations, and defined genetic backgrounds. Recent work has demonstrated that the magnitude of genetic interactions can be influenced by environmental factors [24], and even ploidy level [25]. Yet the generality of such findings remains uncertain. Thus this remains an essential, but poorly explored area of fundamental genetics, as our understanding of epistasis, and our inferences of the topology of genetic networks are often derived from studies of genetic interactions [26]–[33]. In addition, modifier screens have been extremely important, and have identified large numbers of genes that interact to influence the visible expression of the phenotype of the focal mutation, even when the modifier may not have a visible phenotype by itself [34], [35]. We have previously shown that the phenotypic effects of an allele of the *scalloped* gene (*sd^E3^*) in *Drosophila melanogaster* is profoundly influenced by wild-type genetic background (Figure 1B), with effects extending to wing disc transcriptional profiles [36]. One gene that was transcriptionally regulated in a background-dependent matter, *optomotor blind*/*bifid* (*omb/bi*), was then examined in a double mutant combination with *sd^E3^*. We demonstrated that the phenotypic consequence of the interaction between these mutations was markedly influenced by wild-type genetic background. In one wild-type background the double mutant combination resembled the individual *sd^E3^* phenotype, while in the other wild-type background, the *omb* mutation behaved as a strong synthetic enhancer of *sd* [36].

Our findings clearly demonstrate the influence of wild-type genetic background on this genetic interaction, but an important challenge is to determine whether such context dependent effects are widespread. To address this question we performed a genome wide-screen for dominant modifiers of *sd^E3^* using two wild-type genetic backgrounds. Our results suggest that the majority (∼74%) of all modifiers are background-dependent. The background-dependence of the modifier alleles are in part due to the wild-type strains differing in overall sensitivity to mutational perturbations. Using a subset of the deletions spanning the range of phenotypic effects of modifiers, we observed that the interaction effects were consistent using an additional allele, *sd^ETX4^*. Furthermore, we show that the deletion effects are a result of the interaction with mutations at the *sd* locus, and not a simple consequence of haplo-insufficiency in the genomic region of the deletion. We also demonstrate that the background-dependent interactions of modifiers with *sd^E3^* are linked to the same genomic regions that contribute to the background-dependent effects of the allele itself. We argue that the phenotypic expressivity of mutations can be considered a quantitative trait, and a more comprehensive, context-dependent view of the effects of mutations needs to emerge.

## Results

### The majority of dominant modifiers of *sd^E3^* are dependent upon wild-type genetic background

Genetic modifier screens are powerful tools to both identify interacting factors that contribute to signaling networks, as well as to infer their topology. This approach has shaped our understanding of the genetic basis of many traits, across numerous organisms. However little is known about how wild-type genetic background influences genetic interactions. We previously demonstrated that the genetic interaction between mutations in two genes, *sd* and *omb*, is dependent on genetic background [36]. To determine if such an effect is a general phenomenon we performed an analysis of genome-wide genetic interactions between the *sd^E3^* mutation and deletions generated in otherwise isogenic backgrounds spanning the autosomes of *Drosophila*.

We first verified that deletions spanning a number of putative candidate genes (*Dll*, *wg*, *vg*) previously demonstrated to interact with *sd* modify the *sd^E3^* phenotype. In each of these instances the deletions confirmed previous expectations for the interaction (Figure S1B). We then screened the autosomes, with two independent sets of genomic deletions, DrosDel [37] and Exelixis/BSC [38], [39], each generated in an independent isogenic progenitor background (Figure 1B). In total 723 deletion-bearing strains (spanning ∼90% of the autosomal genome) were crossed to *sd^E3^* in each wild-type background. F1 males hemizygous for the *sd^E3^* mutation and heterozygous for the deficiencies were scored.

**Figure 2:**
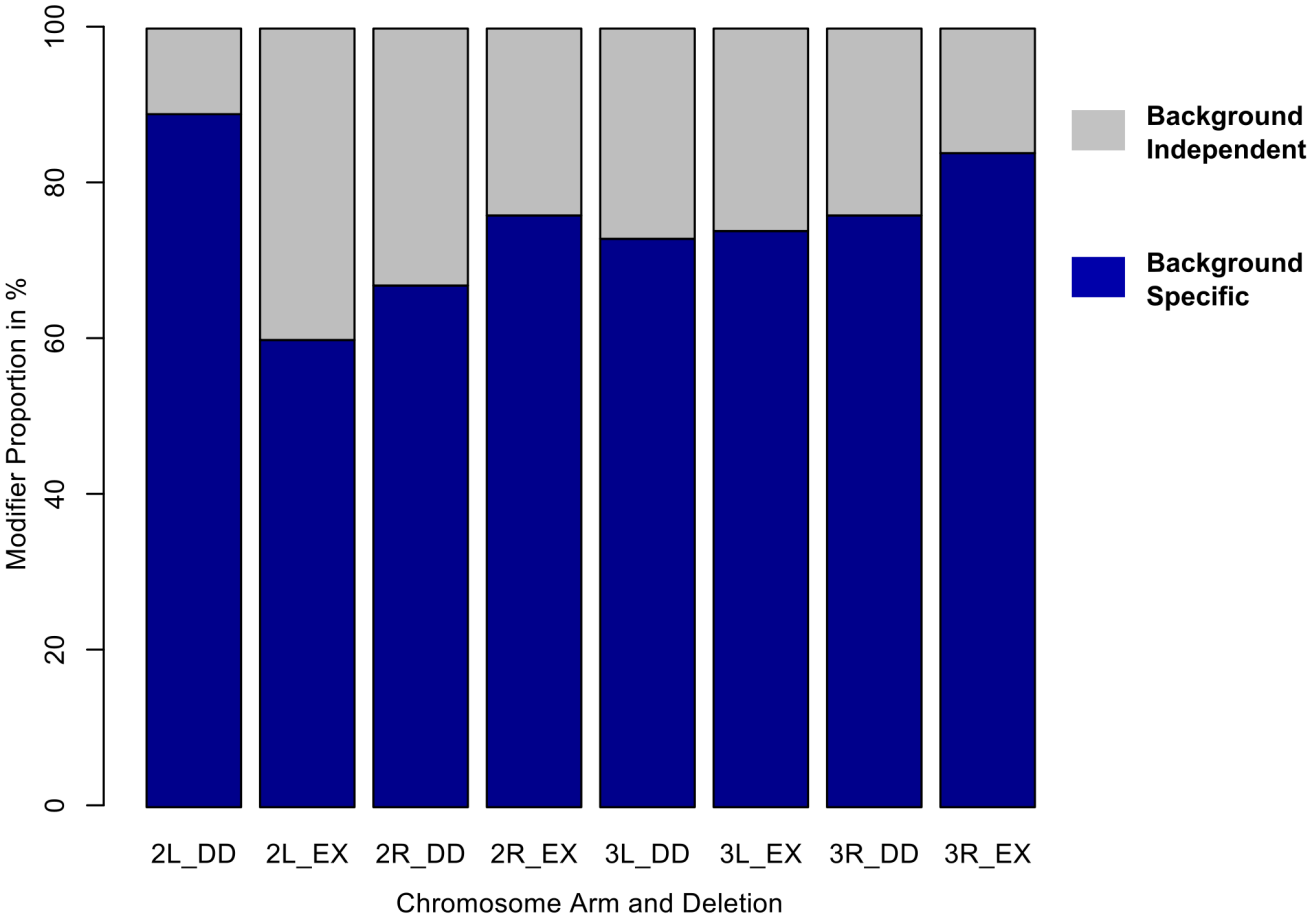
The majority of autosomal modifiers of *sd^E3^* are background-dependent. A) Proportion of deletions that modify the *sd^E3^* phenotype in a background-dependent or -independent manner, by chromosome arm and deletion collection. DD = DrosDel collection. EX = Exelixis collection. Numbers at the bottom of each bar indicate whether the effects are in autosomal chromosome two or three, while the letters L and R represent whether the effects are found on the left or right chromosome arms, respectively.

**Figure 3:**
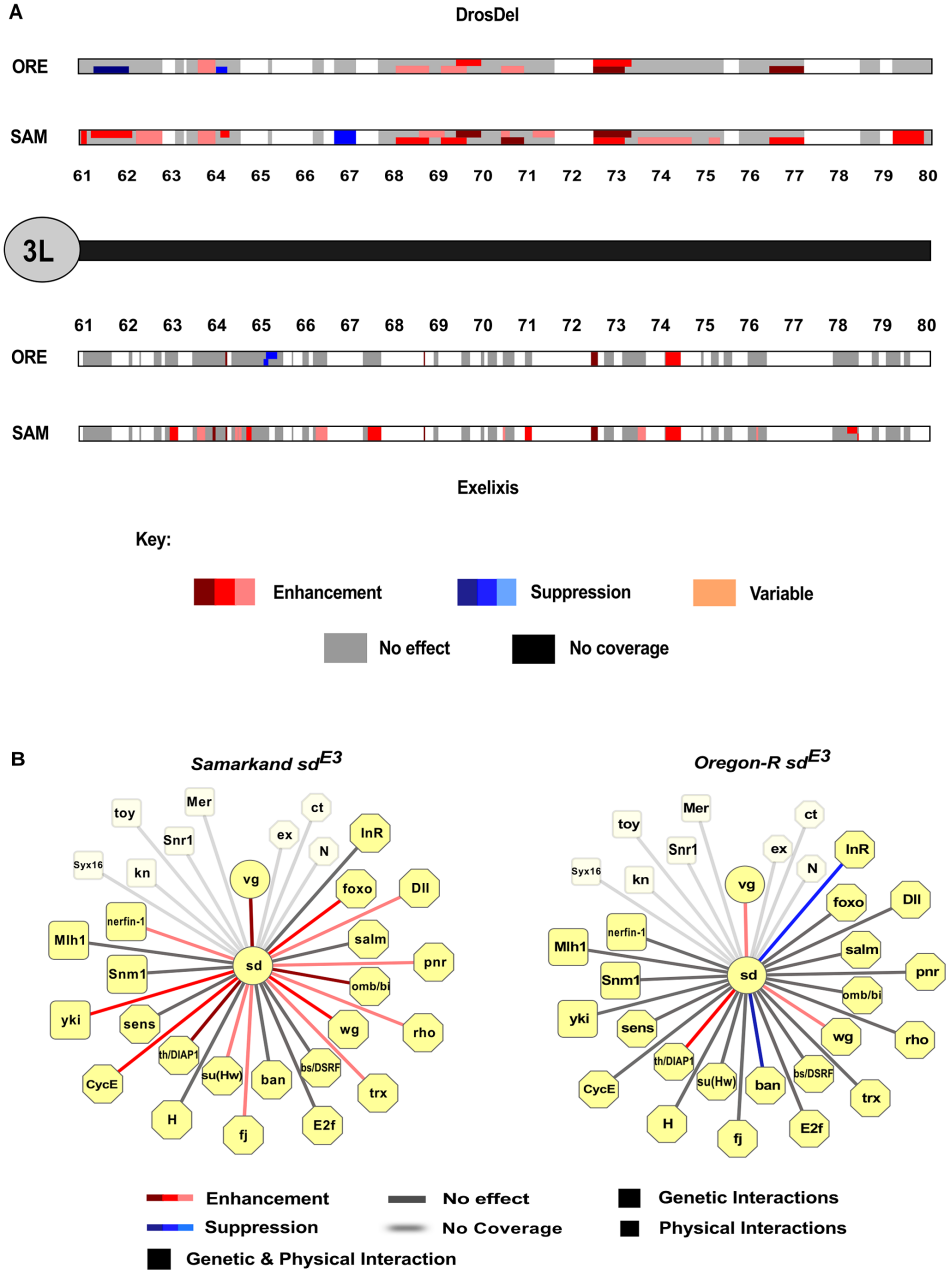
Genomic distribution of background-dependent and independent modifiers of *sd^E3^*. A) Example of the distribution of background-dependent and -independent modifiers of *sd^E3^* on the left arm of chromosome 3 for each deletion collection. The cytological location (61–80) of all deletions on the left arm of chromosome 3 are shown. Regions with no coverage are left blank (white). While there are several locations that show co-enhancement or suppression for Samarkand (SAM) or Oregon-R (ORE), most show an effect in only one background, and occasionally opposite effects (i.e. between 61–62 in DrosDel), consistent with sign epistasis. In a given collection where there were two deletions with overlapping genomic locations (or were nested), the regions in the figure are divided vertically to show the effect of each deletion. The remaining chromosome arms are shown in Figure S4. B) Evidence for background dependent interactions for *apriori* known interacting loci. For deletions that covered the known interacting factors of *sd*, we show the background dependent effects [59]. Unlike the finding for the genome as a whole, there appears to be more synthetic enhancers in Samarkand than Oregon-R.

For the 198 deletion strains that consistently modified the *sd^E3^* wing phenotype, ∼74% of the observed effects were dependent on wild-type (Oregon-R vs. Samarkand) genetic background (Table 1). Frequently, the background contingency was a result of severe effects in one wild-type genetic background, with modest or no effects in the other (Figure 1A and 2, Figure 3A). A complete list of modifier regions, and putative candidate genes can be found in Table S1. An example of the physical location and contribution of these effects is illustrated using the left arm of chromosome 3 (Figure 3, Figure S4), where background-independent and -dependent effects are illustrated, including some deletions with opposing effects in terms of modifying the *sd^E3^* phenotype.

**Table 1:** Number of background dependent and independent modifiers recovered by chromosome arm and deletion collection.

Chromosome arm	2L	2R	3L	3R
Modifier proportion in DrosDel (Background dependent/Total # of modifiers)	8/9 = 89%	10/15 = 67%	22/30 = 73%	19/25 = 76%
Lines Screened	94	39	59	95
Modifier proportion in Exelixis (Background dependent/Total # of modifiers)	20/33 = 60%	19/25 = 76%	17/23 = 74%	32/38 = 84%
Lines Screened	125	82	84	145

Similar results were obtained from the linear model, adjusting for multiple contrasts (see results).

We confirmed these results using a linear model (ANOVA), by asking what proportion of all “significant” modifiers also had a “significant” interaction effect between genetic background and the deletion. Based upon these criteria ∼79% of modifiers demonstrated background dependence. While each cross was carried out independently, there were a large number of crosses performed, and each deletion bearing genotype was compared to a common set of controls from within each block of crosses (see methods). Therefore we utilized several methods that adjust for multiple comparisons. While these methods will decrease the number of deletions deemed modifiers using standard comparisons (i.e. α = 0.05), we are primarily interested in the proportion of such modifiers that are due to background dependent effects. Using False Discovery Rate (FDR) we observed a similar frequency (∼78%) as with unadjusted p-values, while with the sequential Bonferroni (Holm) it was ∼68%. Regardless of the exact approach used, it is clear that the vast majority of modifiers recovered are background dependent.

We performed this screen using two different sets of deletions, each of which varied in the size of the deletion. We observed little association between deletion size and severity of phenotypic modification (Samarkand: correlation-0.09 & -0.08 using Exelixis & DrosDel respectively; Oregon: −0.061 & −0.067 using Exelixis & DrosDel deletions respectively, Figure S5). The lack of association between size of deletion and magnitude of effect suggests that it is unlikely that the observed effects are due to the number of genes perturbed in each deletion.

These key results suggest that at least in sensitization screens, and possibly for many studies of genetic interaction, wild-type genetic background will have profound influences on the range of phenotypes observed and the modifiers that are identified, with only a subset of modifiers being background-independent. Using Flymine and Droid [40], [41] as well as literature mining we examined all of the previously identified genes that act as genetic modifiers, protein-protein interacting partners, or are targets of transcriptional regulation by SD. From these sources we collated evidence for 19 genes that were covered by deletions in this screen (i.e. excluding genes on the X), and all but one (*sens*) were recovered as genetically interacting with *sd^E3^* (Figure 3B). However, more than 50% of these specific loci demonstrated background-specific interactions with *sd^E3^*, including *vg*, which is known to physically interact with SD to form a heterodimer, and is transcriptionally regulated by this complex. Several well-known genetically or physically interacting genes (such as *salm* and *yki*) showed surprisingly mild enhancement of the phenotype, which may be a result of the particular wild-type backgrounds used in this study. These findings suggest that even for well-characterized interacting genes, the influence of genetic background can be substantial, consistent with the flexible nature of genetic interactions. An important caveat to this interpretation is that many of these deletions may contain more than one gene. This could potentially mean that the interaction is due to both the deletion of the focal gene as well as other loci nearby. Yet, as described above, we observed no evidence for a relationship between deletion size and magnitude of effect, suggesting that this may be a minor contributing factor.

### Variation in the extent of epistatic effects is in part due to differences among the wild-types in sensitivity to mutational perturbation

**Figure 4:**
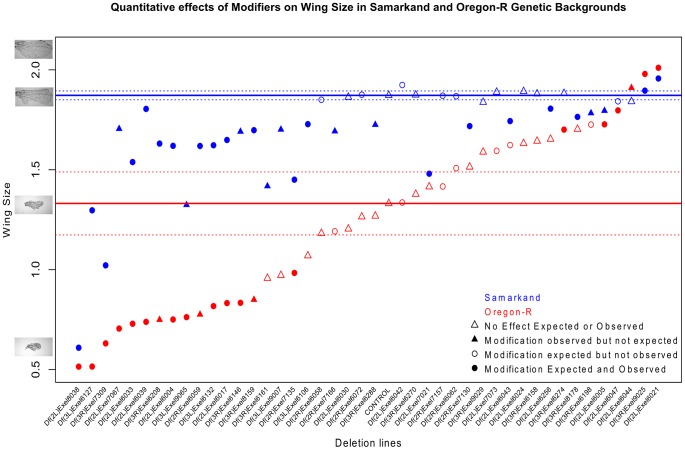
Background dependence is partially a consequence of strain specific sensitivity to genetic perturbation. Quantitative effects of a subset of 44 deletions on the modification of the *sd^E3^* phenotype are shown. The deletions are rank-ordered based on wing size in the Oregon-R background. Enhancement and suppression of the *sd^E3^* phenotype is much greater in the Oregon-R background, relative to Samarkand, in both absolute (shown) and relative terms (not shown). Solid and stippled lines (blue and red) represent the mean and 95% confidence interval, respectively, for wing size in the control *sd* hemizygous males (*sd^E3^*/**Y**). Circles represent deletions with an *a priori* expectation of modification based on the initial semi-quantitative screen, while triangles represent deletions with no observed effect in that screen. Filled symbols represent a significant observed effect in the quantitative screen. The Y axis shows a measure of wing size using centroid size (see methods).

To further validate, refine, and extend our analysis we quantified a subset of 44 of the Exelixis deletion lines that spanned the range of modifier phenotypes across both severity and background-dependence. Interestingly (Figure 4), the background-dependent interactions are clearly a result of both specific differences with respect to the nature of sensitizing mutational effects in each background, as well as to the degree of sensitivity to mutational perturbation. Indeed, the *sd* ^E3^/**Y**; Deletion/+ combinations in the Oregon-R wild-type background demonstrated considerably more variation between deletion strains, compared to the same genotypes in Samarkand (Figure 4). Despite the fact that the *sd^E3^* mutation in the Oregon-R background had more severe loss of wing tissue (Figure 1, Figure S1), the range of both enhancement and suppression exceed that of the same mutation in the Samarkand background (Figure 4). The between deletion co-efficient of variation (CV) for wing size in the Oregon-R background is approximately double that (0.34) of the Samarkand background (0.15). These results were confirmed using a Levene's test with a non-parametric bootstrap. Despite the differences in both degree and spectrum of sensitivity, there was still a moderate correlation of effects of the *sd* ^E3^/**Y**; Deletion/+ combinations (0.66, CI(0.46,0.8)) across the two wild-type backgrounds. These data indicate many of the modifiers are acting in the same direction, although vary for magnitude of effect. Interestingly, even the non-genetic component of phenotypic variation observed for Oregon-R *sd^E3^*/**Y**; +/+ in crosses to the wild-type deletion progenitor shows considerably greater phenotypic variation for wing size compared to Samarkand (Figure 4), although it is unclear if this is related to the changes in within strain variation (robustness).

While the semi-quantitative measure of wing size used for the initial screen, and quantitative measure described above are highly correlated (see methods), a few putative modifier regions failed to replicate in the tertiary validation cross with quantitative measures. Similarly a few deletion lines that were expected to not have an effect (based on the initial screen), did have one with the quantitative measure. However these potential false positives and negatives are few, of similar numbers, and thus are not expected to influence the overall conclusions.

### The influence of the deletions for modifying *sd^E3^* is not correlated with their effect on wild-type wing size

One possible explanation for these results would be that the deletions influenced wing size, *per se*, and the results were not a specific consequence of the interaction between *sd* and the deletion. To investigate this we quantitatively examined females who were heterozygous for the *sd^E3^* mutation and for the deletions (i.e. *sd^E3^*/+ ; Deletion/+) across each genetic background. These females have qualitatively “wild-type” wings, and previous work did not observe an effect of *sd^E3^* on wing size in females as heterozygotes [42] (although it did influence wing shape). Therefore we quantified these females across the same set of deletions as described above. If the deletions were not generally acting as modifiers of the “sensitized” *sd* mutant phenotype in hemizygous males, but as general modulators of size, then we would expect a strong positive correlation between the effects on size in males and females (*sd^E3^*/+ ; Deletion/+ vs. *sd^E3^*/**Y** ; Deletion/+). The correlation between Samarkand and Oregon-R *sd^E3^*/+ ; Deletion/+ females was ∼0.8, suggesting that the effects of the deletions on overall wing size is similar across backgrounds. However the correlations within each background (i.e. *sd^E3^*/+; Deletion/+ vs. *sd^E3^*/**Y** ; Deletion/+) were 0.22, (CI −0.08, 0.49), and 0.21, (CI −0.08, 0.48) respectively, and neither case was significantly different from 0. The lack of a correlation indicates that the influence of the deletions in *sd^E3^* hemizygous males is largely independent of any effects on overall wing size. More importantly the CV for wing size in females (across deletions) for both backgrounds was ∼0.03, which is 5× and 10× less than that observed for *sd^E3^* hemizygotes in Samarkand and Oregon-R respectively (Figure S6). This suggests that most of the phenotypic variation for wing size due to the deletion is observed when the backgrounds are “sensitized” with the *sd* mutation, while having relatively little influence on wild-type wing size.

### Loci influencing background dependent interactions are linked to those influencing phenotypic expressivity of *sd*

Are the loci influencing the background-specific genetic interactions the same as those that modulate phenotypic expressivity for wing size of the focal *sd* ^E3^ mutation? To address this question we generated a set of backcross lines between Oregon-R and Samarkand (both fixed for *sd^E3^*), where “long” wings were selected in the backcross to the Oregon-R background, and “short” wings in backcrosses to the Samarkand background (Figure S3). Using ∼30 SNPs polymorphic across backgrounds, we verified that these backcross lineages showed expected genotypes for more than 90% of markers (i.e. phenotypically short wings but with Samarkand genotypes). Among the molecular markers that did introgress, include those tightly linked to the unknown causal loci on 2R near cytological band 48 and at the centromere of 3L [36]. If the loci modulating the magnitude of the genetic interactions were caused by genes other than those influencing the background-specific disruption of wing development, we would predict weak correlations between *sd^E3^*/**Y**; Deletion/+ in Oregon-R and the equivalent genotype from the “short” backcross (with an otherwise Samarkand background). Similar logic prevails for the Samarkand and the “long” phenotype. However, even using semi-quantitative measures, it is clear that these are highly correlated; 0.82 (CI 0.66–0.91) and 0.86 (CI 0.73–0.93) respectively. These results are consistent with the loci influencing the background-dependent genetic interactions being the same as those influencing the background-dependent effects on the phenotypic expressivity of the focal *sd* ^E3^ mutation.

### Background dependent interactions are consistent across additional alleles of *sd*

The results described above demonstrate that the loci that influence the background dependent nature are linked to those influencing phenotypic expressivity of the mutation itself. However, it was unclear if the observations were due to some particular properties of the *sd* ^E3^ allele, or a more general function of perturbation at the *sd* locus. To address this, we retested a subset (29) of the deletions spanning the range of phenotypic effects with *sd^E3^*, using an additional allele *sd^ETX4^*, across each genetic background. The phenotypic consequences of *sd^ETX4^*, while background-dependent, are somewhat weaker than *sd* ^E3^ (Figure S7A). Despite these phenotypic differences, there was a moderate to high correlation across the modifiers' effects on these two alleles. In the Oregon-R and Samarkand wild-type genetic backgrounds respectively, the correlation between the effects of the deletions on the phenotypes of the *sd^E3^* and *sd^ETX4^* allele was 0.66 (CI 0.38–0.82), and 0.76 (CI 0.55–0.88). In addition the general pattern of greater sensitivity to mutational perturbation by modifiers of the *sd* phenotype appears to be generally maintained (Figure S7B). These results demonstrate that even across multiple alleles, the background dependence of the modifiers is maintained.

### *vestigial* (*vg*) interacts with *sd* in a background dependent manner

Although the primary goal of this study was to explore the flexibility in genetic interactions, not to identify candidate genes, for confirmatory purposes, we examined several genomic regions that demonstrated background-dependent or -independent modifiers (Table S2). Interestingly, one region, 49E1, contained *vg*, which encodes a SD-regulated transcriptional factor that forms a heterodimer with SD. Fine mapping, followed by the use of candidate insertional mutants (co-isogenic to the Exelixis deletions) confirmed that the *vg^F02736^* allele behaved as a background-dependent enhancer with strong enhancement in Samarkand, but very weak enhancement in Oregon-R. We followed this up by introgressing this allele into both the Samarkand and Oregon-R background. Again we observed background-specific enhancement of the *sd* phenotype. Other fine mapping regions suggest several candidate genes, although for at least one region, no obvious candidate gene could be determined (Table S2).

## Discussion

Genetic modifier screens have provided an indispensible tool for identifying interacting sets of genes, providing a glimpse into the underlying genetic network, and a point of entry for further molecular characterization. Much of our knowledge of network topology has depended on the use and interpretation of such genetic interactions [43], and such information is included in many common databases and graphical representations of networks such as in FlyMine and DroID [40], [41] as well as flybase [44]. The importance of modifier screens cannot be over-stated for the identification of interacting genes. Yet the generality of networks defined by these interactions is unclear, given that such screens (and thus the nature of the interactions) are generally performed in isogenic wild-type backgrounds to prevent numerous artefactual findings. In this study, we demonstrate that the majority of such genetic interactions are dependent on wild-type genetic background. Our results suggest that different wild-type strains vary in their general sensitivity to mutational perturbation, as well as having strain specific responses to such modifiers (Figure 3, Figure 4, Figure S4). Both of these factors contribute to both quantitative and qualitative changes in the observed phenotypic effects across the focal *sd* mutations and the deletions. While the majority of the observed background dependent effects changed the magnitude of the interaction, we did observe several instances of sign epistasis, where the deletion modified the phenotypic expressivity of the *sd* allele in opposite ways across the different backgrounds. This genotypic conditionality suggests that genetic networks may be quite flexible, with segregating variation in natural populations influencing magnitude and possibly sign of interactions. Indeed, such context dependence in genetic interactions, whether due to genetic background, or other factors needs to be recognized as a likely general phenomenon.

It is probable that the results presented here under-estimate the degree of background dependent genetic interactions. In this study we screened for dominant modifiers of the *sd* mutations, and only two wild-type strains were used heterozygous against common isogenic tester strains. It is to be expected that double mutant combinations in each homozygous genetic background would demonstrate even more background dependence from the phenotypic expression of recessive alleles, as has been examined for particular pairs of interacting loci in a few model systems [4], [23]. Yet in this relatively simple design, ∼74% of modifiers were background-dependent (Figure 2A, Figure 3A). Even for functionally characterized genes that interact with *sd*, over 50% demonstrated interactions that were background-dependent (Figure 3B). The results were consistent both across multiple alleles of *sd* (Figure S7), and across backcross-introgression lines (Figure S3). In addition the results were consistent when we moved from particular deletions to individual mutations. The well-known interacting factor *vg* demonstrated background-specific interactions from the segmental deletion containing it, to an individual mutation in the gene, with strong enhancement in Samarkand but mild effects in Oregon-R, similar to previous observations between *sd* and *omb* [36].

Overall, the observed background-dependence was due to a combination of both sensitivity of the wild-type background to mutational perturbation, as well as specific patterns of interactions between deletions and the *sd^E3^* mutation across backgrounds. Despite the principal effect of *sd* ^E3^ being more severe in Oregon-R than in Samarkand, both the suppressors and enhancers recovered were also of greater magnitude in the Oregon-R background (Figure 4). The choice of a particular wild-type background for sensitization screens could lead to profoundly different interpretations with respect to the number and nature of modifiers recovered. This is of some concern when it is acknowledged that wild-type strains with the same names may not be genetically identical across different labs due to new mutations, bottlenecks, recombination and contamination. Thus the inferences made from studies of pairwise mutational interactions may be difficult to generalize, and may in part explain why the same allelic combinations can result in different phenotypic outcomes. In this study, it was not just change in magnitude of the genetic interactions, but in some instances the sign (i.e. enhancer vs. suppressor) of the interaction that was contingent on the genetic background. Such findings may explain why attempts to replicate findings of genetic effects (such as GWAS) can be difficult. Despite the obvious complications, the background-dependent nature of these effects has a beneficial aspect; new loci can be identified by performing modifier screens in additional wild-type backgrounds. Indeed with many wild-type strains being sequenced to perform genome wide associations, this may provide an additional tool for rapid identification of new interacting loci. Additionally, the use of RNAi across multiple genetic backgrounds may be able to facilitate such studies [45]. However leveraging such complex genetic interaction data may require a new population level framework to interpret the results.

### What is the genetic architecture underlying the background dependent interactions?

There are outstanding questions that our study is unable to address. The background dependent nature of the genetic interactions could be the result of a “third-order” effect between the *sd* mutation, the hemizygous allele uncovered over the deletion and other loci across each wild-type genetic background. An alternative, and perhaps simpler explanation would be of differential quantitative complementation uncovered by the deletion [46]. In such cases, the variation in the degree of the modification of the focal mutation (*sd*) is a direct result of the alleles that differ across backgrounds uncovered by the deletion. While we expect that our results are a combination of both explanations, it is likely that without very high resolution mapping of the genomic regions, or test of specific polymorphisms will we be able to determine the relative contribution of each type of interaction. However the previous work that motivated this current study, namely the background dependent interaction between *sd* and *Omb* was clearly due to a third order effect [36]. Understanding the degree to which increasingly higher order epistasis contributes to phenotypic variation is under-explored but of great importance [47].

One curious finding of our study was that the background (Oregon-R) that demonstrated the higher degree of phenotypic expressivity of the focal *sd* mutations, showed increased sensitivity to mutational perturbation (both enhancers and suppressors) as well as greater phenotypic variation within strain. Recent work has demonstrated that loci can influence trait variability (“noise”) directly [48]–[50], including naturally occurring variants in the *Hsp90* gene of *Drosophila* [51]. Indeed even cell-to-cell variation, and variation in penetrance appears to have a complex genetic architecture [48] influenced by variability in gene expression [52]. It is unclear whether the loci that contribute to increased phenotypic “noise” also contribute to the amplified sensitivity to mutational perturbation as seen in the Oregon-R vs. Samarkand wild-type backgrounds. In previous work Oregon-R does have higher levels of phenotypic variation in quantitative measures of wing shape, but no increased sensitivity to weak (heterozygous) mutational perturbation [42]. However the focal mutations used in the current study (*sd^E3^* and *sd^ETX4^*) represented more severe perturbations to wing development, so this may not provide an adequate comparison. Regardless, this remains an unanswered question, and a potential link between so-called variance controlling genes and sensitivity to perturbation would have important implications for the genetic architecture of canalization and robustness [5], [53].

One constraint of the current study is that we utilized a hypomorph of moderate phenotypic effect, as opposed to a null allele. While a formal definition of functional epistasis (sensu [54]) requires the use of null alleles, most interaction screens utilize alleles of comparable (hypomorphic) effect to allow the recovery of both enhancers and suppressors. Nevertheless, previous work has demonstrated that null alleles can also show background-dependence effects in the primary effect of the mutation, including on development, growth and viability [1], [2], and our results demonstrate that these conditional effects are likely to be reflected in the genetic interactions between mutations as well. In addition we demonstrated that the quantitative effects we observed with the interaction between *sd^E3^* and segmental deletions in each wild-type genetic background were correlated when observed across another (weaker) allele, *sd^ETX4^*, suggesting that such effects are not due to a particular allele. We also demonstrated that the effects of these interactions are tightly linked to the same genomic regions that contribute to the primary background-dependent phenotypic effects of the mutations. Thus for our system, the genetic variants influencing the phenotypic expressivity of the focal mutation appear to be the same as those modulating both the magnitude, and potentially the sign of genetic interactions between mutations.

While the positive and negative implications for modifier (and other genomic) screens is clear, the potential flexibility of genetic networks given segregating variation in a population needs to also be considered. In particular an allele entering a population (either as a new mutation, or as a result of introgression from another population or species) may not have a “fixed” effect on fitness; instead the genetically contingent effects of the allele result in a distribution of phenotypic effects, including a possible change in sign (i.e. from deleterious to beneficial).

## Materials and Methods

Data and scripts associated with this manuscript are located on DRYAD: http://dx.doi.org/10.5061/dryad.4dt7c

### Fly stocks

The Oregon-R strain was originally obtained from the Bloomington stock center, while Samarkand was obtained from the lab of Dr. Trudy Mackay. For both strains, we further inbred them to near isogenicity, and tested via a panel of 30 polymorphic markers to confirm there was no contamination or residual heterozygosity. A combination of sequencing and PCR-based genotyping suggests that these two strains have an approximately 2% divergence from one another, and that all sequenced regions examined to date are a subset of variation from natural populations. The X-linked *sd^E3^* mutant allele (obtained from the Drosophila stock center, Bloomington IN), used in this study is caused by a P{*w*[E] *ry*[1t7.2] = wE} transposon located in the third intron of the *sd* gene [55]. This mutant allele was introgressed into two lab wild-type strains, Oregon-R and Samarkand, both marked with *white (w)*, by repeated backcrosses involving homozygous mutant female and the wild type male for over 20 generations [36]. These lines have been subjected to extensive genotyping to verify the extent of the introgression, and to avoid contamination. The *sd^ETX4^* and *vg^F02736^* alleles were also obtained from the Bloomington stock center, and were introgressed for 20 generations into each wild-type strain.

#### Deletion lines (obtained from Bloomington stock center)

We utilized the DrosDel [37] and Exelixis/BSC [38] collections of lines that have defined segmental deletions collectively spanning ∼90% of the autosomes, with an average deletion size of 400 kb and 140 kb respectively. Deletion panels were generated in isogenic backgrounds and include overlapping as well as nested deletions within and between each panel. The progenitor wild-type strains (one for DrosDel & one for Exelixis/BSC) were used in crosses to generate background-specific control flies. While spontaneous loss of the tip of chromosome 2L, containing *l(2)gl* could potentially confound the results of our screen [56], our tests of a subset of these deletions did not demonstrate non-complementation with *l(2)gl*. Thus it is unlikely that this is a confounding factor in our analysis.

### Dominant modifier screen

#### Crosses

To assess the influence of wild-type background on genetic interactions we used a dominant modifier screen, and examined *sd* mutant hemizygotes who were heterozygous for the deletions. Deletion lines (see above), and their isogenic wild-type progenitor strains were crossed to homozygous *sd^E3^* mutant females (Figure 1). Flies were allowed to mate and lay eggs for 3–4 days and then transferred into fresh vials for a backup. All crosses were performed at 24°C 65% RH on a 12∶12 hr light∶dark cycle in a Percival (Model: I41VLC8) incubator. For each deletion, *sd^E3^*/**Y**; Deletion/+ male progeny were scored in each genetic background (Oregon-R and Samarkand) for enhancement or suppression of the *sd^E3^* phenotype (Figure 1A). Thus we scored flies hemizygous for *sd*, and heterozygous for the deletions. Deletion crosses were performed in large blocks, involving 25 to 100 deletions per block (paired across backgrounds), and for each block a simultaneous set of control crosses with the progenitor wild-type strains for DrosDel and Exelixis flies was also performed. Nevertheless, there was negligible variation in the wing phenotypes of the flies resulting from the control crosses across all the blocks (not shown). However, for appropriate inferences, phenotypic analysis for all crosses within a block were made with respect to specific sets of control crosses from within that same block. We screened between (5–20) flies for each cross (crosses with fewer than 5 progeny were re-tested), with a mean/median of 8.2/7 flies per cross. Any deletion that showed evidence for modification (see below) of *sd^E3^* was re-tested (new crosses) to verify the phenotypic effects. Crosses performed with DrosDel deletions on chromosome arm 3L showed a marked increase in the number of modifiers relative to other arms (22/59 compared to 37/228 for the rest of the chromosome arms for the DrosDel collection). Thus putative modifiers on 3L were re-tested 3 times each, with consistent results, suggesting that these modifiers are unlikely due to a sampling artefact. In total 723 deletions were tested, with 18,167 flies scored.

#### Scoring technique

For initial assessment of phenotypic modification we performed a semi-quantitative analysis similar to that used by other investigators [57], grouping the progressive loss of wing tissue based on shape and size (proxy for severity of mutation) into 10 categories from A through J (nominal scores of 1–10) such that, category “A” represented a wild type wing phenotype and “J” represented a severely reduced wing phenotype (Figure S1). Pure Samarkand *sd^E3^* individuals were generally category D while Oregon-R *sd^E3^* individuals were category H, with relatively minor variation in these scores. The rationale for such a semi-quantitative approach was two-fold. First, we wished to mirror the genetic screen approaches used in many functional genetic studies (using qualitative or semi-quantitative measures), and second this allowed us to screen a much larger panel of lines. As discussed below, these semi-quantitative measures correlated well with quantitative measures of wing size.

To mimic a traditional genetic screen we assessed interactions based largely on non-overlap distributions of phenotypes, comparing genotypes bearing deletions to their co-isogenic wild-types. While this likely underestimates the number of true interactions of the deletions with *sd^E3^*, it was done so that the observed effects were of an almost qualitative nature (as is often done for visual screens). As discussed above, all putative modifiers were verified at least once with an independent replication cross.

In addition, we also utilized a more quantitative approach, fitting the data to the following linear model:
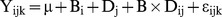
where Y is the semi-quantitative measure of size (1–10), B is the wild-type genetic background (Oregon-R and Samarkand) and D is the deletion (deletion bearing chromosome, or co-isogenic wild-type). We evaluated the results from the linear model. While each cross was performed independently, given that so many crosses were performed, we examined the results (with respect to significant “hits”), with unadjusted p-values, as well as using several methods to control for multiple comparisons (FDR and Holm/Sequential Bonferroni). The analysis was performed using the lm function and p.adjust in R (V 2.12).

### Quantification of size and shape

To validate the primary findings of this study, we repeated crosses, and quantified wing size for a subset of 44 deletions, spanning the direction and magnitude of effects (background dependent-independent, suppressor-enhancer, as well as negative controls) observed in the genome-wide screen. A single wing from each of 5 male flies (*w sd^E3^*/**Y**; Deletion/+) was dissected and mounted in glycerol, for both backgrounds. For the isogenic wild-type control strain, 30 individuals were used from each background-specific set of crosses to better ascertain the degree of variability. Images of the wings were captured using an Olympus DP30BW camera mounted on an Olympus BW51 microscope. Six landmarks (Figure S2) were digitized using tpsDIG software [58] and centroid size was used as a measure of wing size. The landmarks were specifically chosen as they could be discerned on all wings (Figure S2). To quantitatively verify the background-dependent effects of a given deletion on wing size (Figure 4) the following model was used:
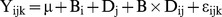
where Y is the Centroid Size, B is the background and D is the deletion. The analysis was performed using the lm function in R (V 2.12) and 95% confidence intervals were constructed using confint. Significance was determined by non-overlapping confidence intervals with controls.

The quantitative measure of wing size used for this analysis, correlates well with the semi-quantitative method and results used for the initial screening (r = 0.82, CI:0.69–0.9 in Oregon-R, r = 0.78, CI:0.63–0.87 in Samarkand). This suggests high repeatability of the initial screen, as well as the semi-quantitative measure of wing size.

To ascertain whether there was a commensurate effect of the genomic deletions in “wild-type” wings (as opposed to the mutant phenotype caused by *sd* mutants), we quantified wing size in females heterozygous for the focal *sd^E3^* mutation with each deletion (*w sd^E3^*/*w sd^+^*; Deletion/+) digitizing the same 6 landmarks on the wing.

### Generation and crossing of “large-wing” and “small-wing” backcross lines of *sd^E3^*

Potentially the genomic regions (from the wild-type strains) that influence the genetic interaction between the deletions and *sd* ^E3^ could be independent of those regions that influence the variation for phenotypic expressivity of the *sd* ^E3^ mutation itself. To test this we generated lines that had “high expressivity” *sd^E3^* phenotypes in an otherwise “low expressivity” background (Figure S3). A backcross-selection procedure was used to introgress the modifiers that contribute to the “large wing” phenotype from the Samarkand background into the “small wing” background of Oregon-R and vice-versa (Figure S3). Upon generation of these lines, we repeated the dominant modifier screen as described above using a subset of 32 of the 44 confirmed modifiers and negative controls. These lines were used in identical crosses to those outlined above, with *sd^E3^*/**Y**; Deletion/+ individuals examined.

### Fine scale mapping

To narrow down several genomic regions to a set of a few candidate genes we utilized an additional set of overlapping deletions in DrosDel, Exelixis and BSC strains followed by use of P-element insertional mutations co-isogenic with the Exelixis panel of lines. We utilized this approach for four genomic regions (49E1, 57B3-B5, 63F2-F7, and 86E13-E16) detailed in Table S2.

## Supporting Information

Figure S1 Scoring scheme and positive controls for *sd^E3^* modifier screen. A) The Semi-Quantitative Scoring Scheme used for the primary screen for the modifiers of *sd^E3^*. The semi-quantitative scoring scheme used for this study was similar to other ones previously used (see methods), allowing for rapid phenotyping of the wings. A comparison of quantitative and semi-quantitative methods with a test data set were highly correlated (not shown). B) Reaction norms from deletions uncovering known interacting genes with *sd*.
(TIF)

Figure S2 Landmarks used to quantify wing size. To quantify wing size in this study we utilized the centroid size calculated from 6 landmarks. These landmarks could be unambiguously found in all specimens that we examined in this study. It is worth noting that for mutations (not used in this study) that influence wing development more severely, these 6 landmarks could not be scored (not shown).
(TIF)

Figure S3 Backcross-selection procedure across wild-type backgrounds with *sd^E3^* to introgress “long” and “short” alleles. The alleles that contribute to the background dependence of the genetic interactions between *sd^E3^* and the autosomal deletions could potentially be the same as those that contribute to the variation in expressivity in the *sd* phenotype. If this hypothesis is false, then we would predict no association between the genomic regions that contribute to variation for *sd* expressivity and the nature of genetic interactions across backgrounds. To test this, we utilized a backcross-selection procedure to move the genomic regions conferring “long” wings into an otherwise “short” Oregon-R background. Individuals from the Samarkand and Oregon-R background bearing the *sd^E3^* allele were crossed together, and F1 flies were mated *interse* to produce an F2 population segregating alleles influencing the expressivity of the *sd* wing phenotypes. Flies with the largest wings (most Samarkand *sd* ^E3^ like) were then crossed to Oregon-R *sd^E3^* individuals, as well as the reciprocal for the shortest wings (crossed to Oregon-R). This two generation procedure was repeated for 12 cycles for the flies being selected for “short” wings, and 19 cycles for those for the “long” wings. This approach allows for the introgression of the alleles influencing *sd* expressivity from one background to the other. A panel of 30 SNP markers known to be polymorphic between Oregon-R and Samarkand were then used to verify the extent of the introgressions.
(TIF)

Figure S4 Distribution of modifiers on remaining chromosome arms. Figure legend and description as for figure 3A. A) Chromosome arm 2L. B) Chromosome arm 2R. C) Chromosome arm 3R.
(PDF)

Figure S5 No association between size of the genomic deletion and magnitude of effect as a modifier of *sd^E3^*. To determine whether the deletions generally uncovered a single or multiple modifier alleles of *sd^E3^*, we examined the relationship between the magnitude of the effect of the deletion on the wing phenotype, and the size of the deletions (in kbp). As seen in these figures, there is no association between them, suggesting that across the set of screened lines, each deletion is likely only uncovering a single modifier allele. However particular individual deletions may have more than one modifier, and modifiers that act in opposite directions.
(TIF)

Figure S6 The effects on wing size of 44 deletions in females heterozygous for *sd^E3^*. To determine the extent of the phenotypic effects of the genomic deletions on wild-type wing sizes, we examined the effects of 44 of the deletions (the same ones used for Figure 4) in *sd^E3^*/+; Deletion/+ females in each background. While the mean wing size differed across wild-type backgrounds, the range of phenotypic effects around each mean was similar (see text). Importantly, the coefficient of variation across strains was ∼10× smaller for wing size for wild-type wings, than for the wings of *sd^E3^* hemizygous males.
(TIF)

Figure S7 The background dependent effects on the *sd^ETX4^* allele. To determine whether the findings observed for the background dependence of the genetic interactions of the *sd^E3^* allele with the deletions would hold across other alleles, we introgressed an additional allele, *sd^ETX4^*, into both Samarkand and Oregon-R, and re-examined a subset of the deletions. A) *sd^ETX4^* also shows profound background dependence with respect to the expressivity of the *sd* phenotype. As described in the text, the results were significantly correlated across alleles. Interestingly the background dependent expressivity of *sd^ETX4^* is substantially weakened in crosses with the Exelixis Deletion progenitor strain. However, the background dependence of the genetic interactions appears to be at least as extreme as that observed for *sd^E3^* (B).
(TIF)

Table S1 Candidate modifier genes (.xls) from the deletion mapping.
(CSV)

Table S2 Results from fine deletion mapping.
(DOCX)
