# Supplementary figures and images for: The Conditional Nature of Genetic Interactions: The Consequences of Wild-Type Backgrounds on Mutational Interactions in a Genome-Wide Modifier Screen
Source: PLoS Genet. 2013 Aug 1;9(8):e1003661. doi: 10.1371/journal.pgen.1003661 (PMC3731224; doi:10.1371/journal.pgen.1003661)

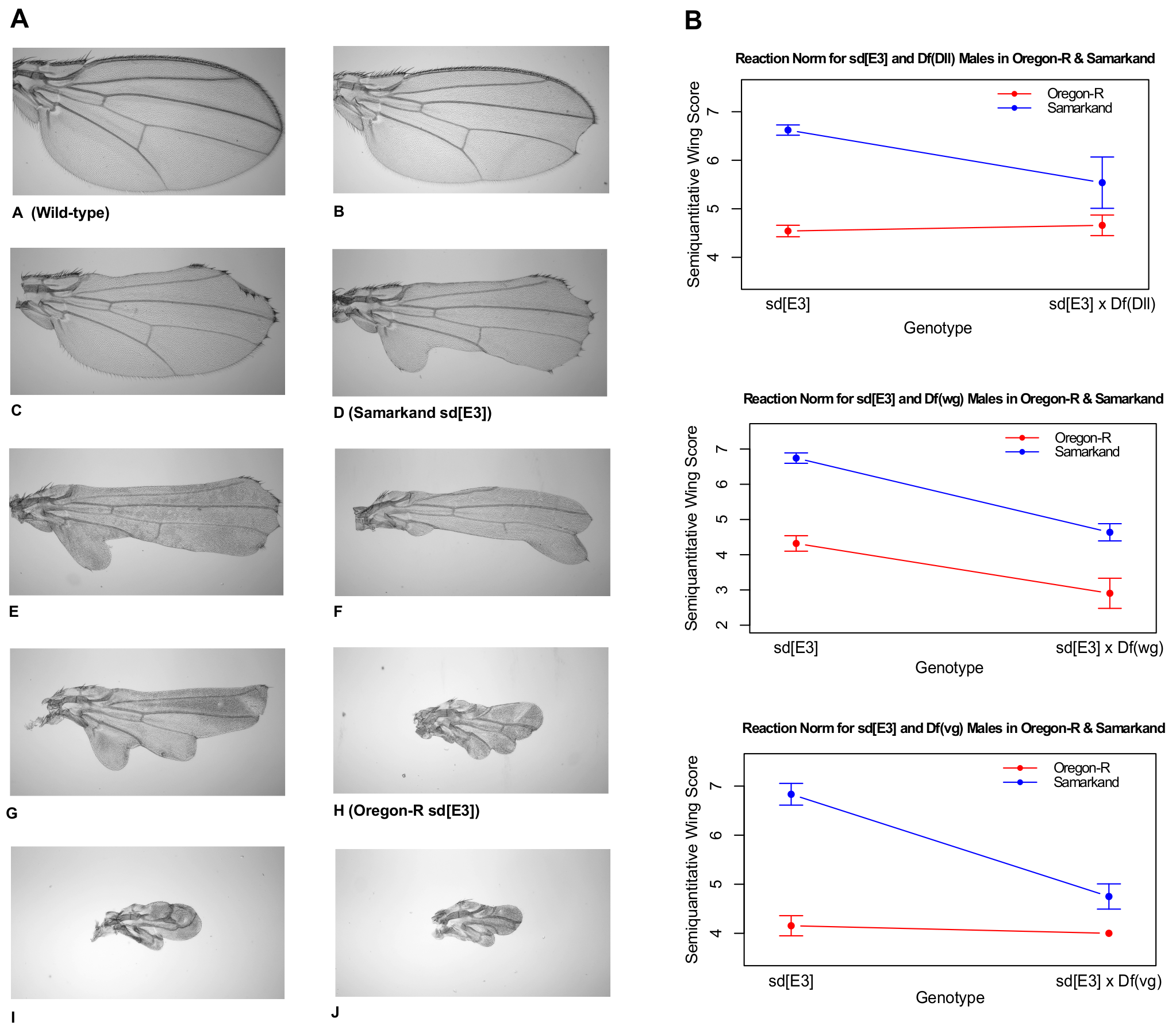

Supplement: Figure S1 — Scoring scheme and positive controls for sdE3 modifier screen. A) The Semi-Quantitative Scoring Scheme used for the primary screen for the modifiers of sdE3. The semi-quantitative scoring scheme used for this study was similar to other ones previously used (see methods), allowing for rapid phenotyping of the wings. A comparison of quantitative and semi-quantitative methods with a test data set were highly correlated (not shown). B) Reaction norms from deletions uncovering known interacting genes with sd. (TIF) [file pgen.1003661.s001.tif]

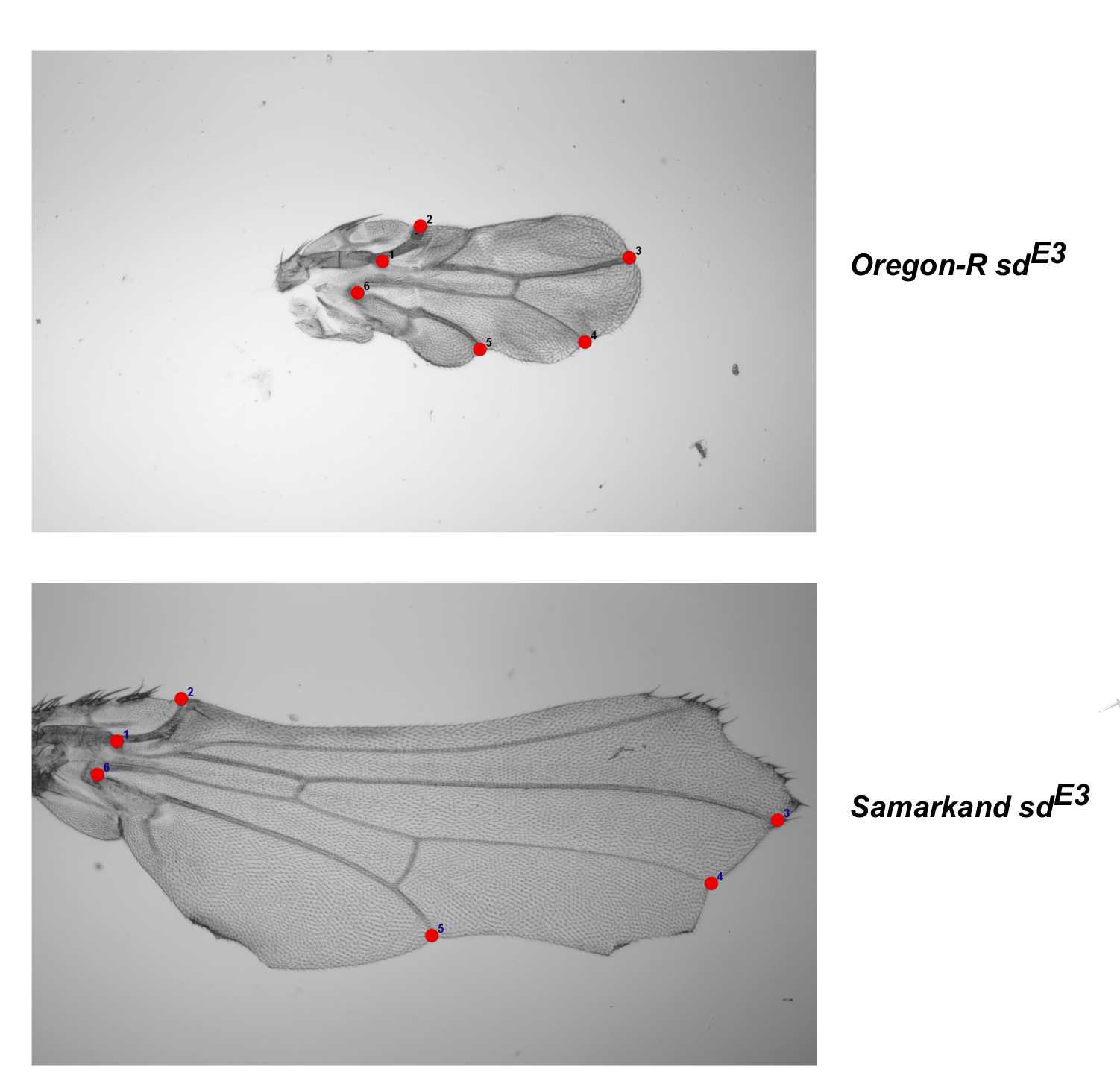

Supplement: Figure S2 — Landmarks used to quantify wing size. To quantify wing size in this study we utilized the centroid size calculated from 6 landmarks. These landmarks could be unambiguously found in all specimens that we examined in this study. It is worth noting that for mutations (not used in this study) that influence wing development more severely, these 6 landmarks could not be scored (not shown). (TIF) [file pgen.1003661.s002.tif]

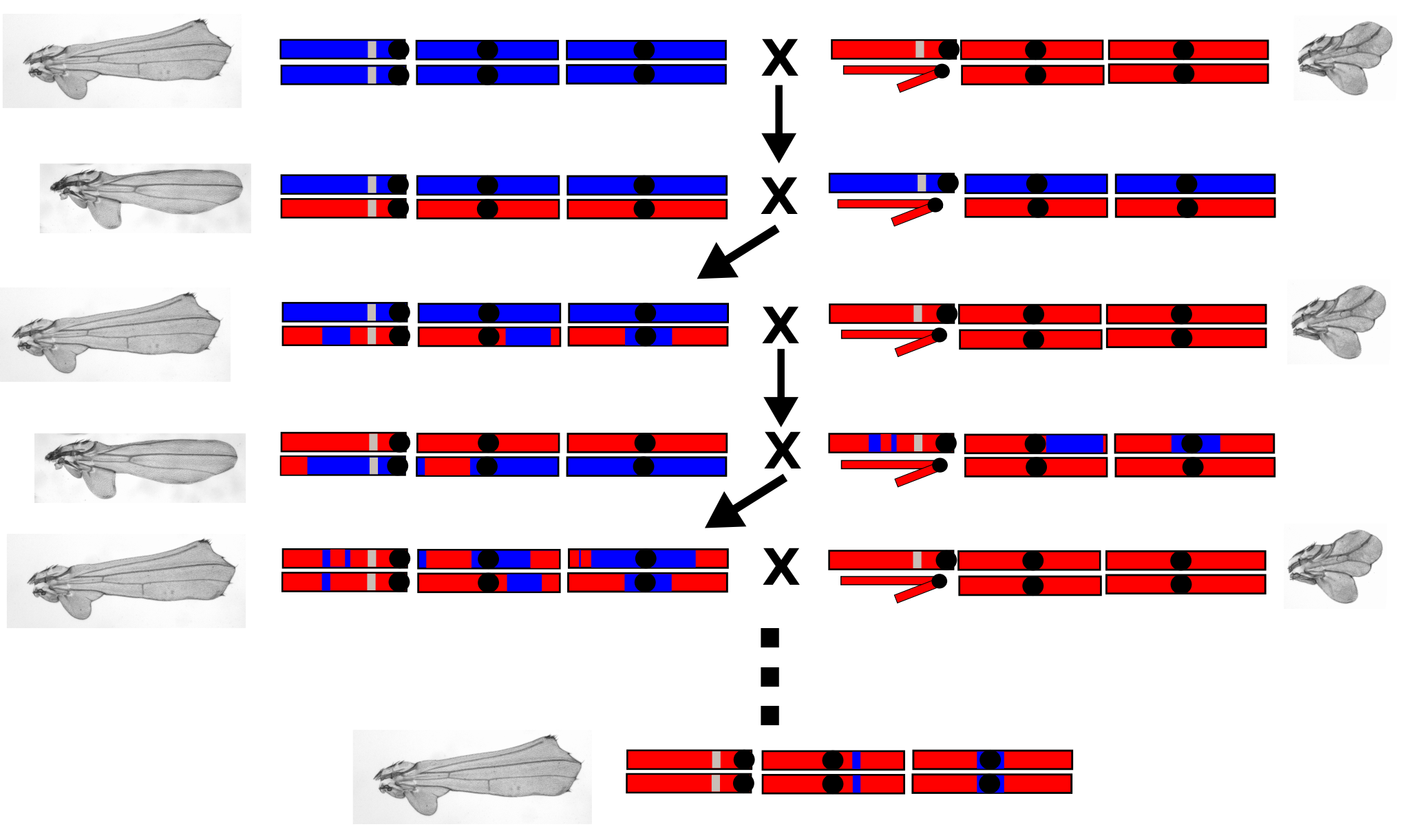

Supplement: Figure S3 — Backcross-selection procedure across wild-type backgrounds with sdE3 to introgress “long” and “short” alleles. The alleles that contribute to the background dependence of the genetic interactions between sdE3 and the autosomal deletions could potentially be the same as those that contribute to the variation in expressivity in the sd phenotype. If this hypothesis is false, then we would predict no association between the genomic regions that contribute to variation for sd expressivity and the nature of genetic interactions across backgrounds. To test this, we utilized a backcross-selection procedure to move the genomic regions conferring “long” wings into an otherwise “short” Oregon-R background. Individuals from the Samarkand and Oregon-R background bearing the sdE3 allele were crossed together, and F1 flies were mated interse to produce an F2 population segregating alleles influencing the expressivity of the sd wing phenotypes. Flies with the largest wings (most Samarkand sd E3 like) were then crossed to Oregon-R sdE3 individuals, as well as the reciprocal for the shortest wings (crossed to Oregon-R). This two generation procedure was repeated for 12 cycles for the flies being selected for “short” wings, and 19 cycles for those for the “long” wings. This approach allows for the introgression of the alleles influencing sd expressivity from one background to the other. A panel of 30 SNP markers known to be polymorphic between Oregon-R and Samarkand were then used to verify the extent of the introgressions. (TIF) [file pgen.1003661.s003.tif]

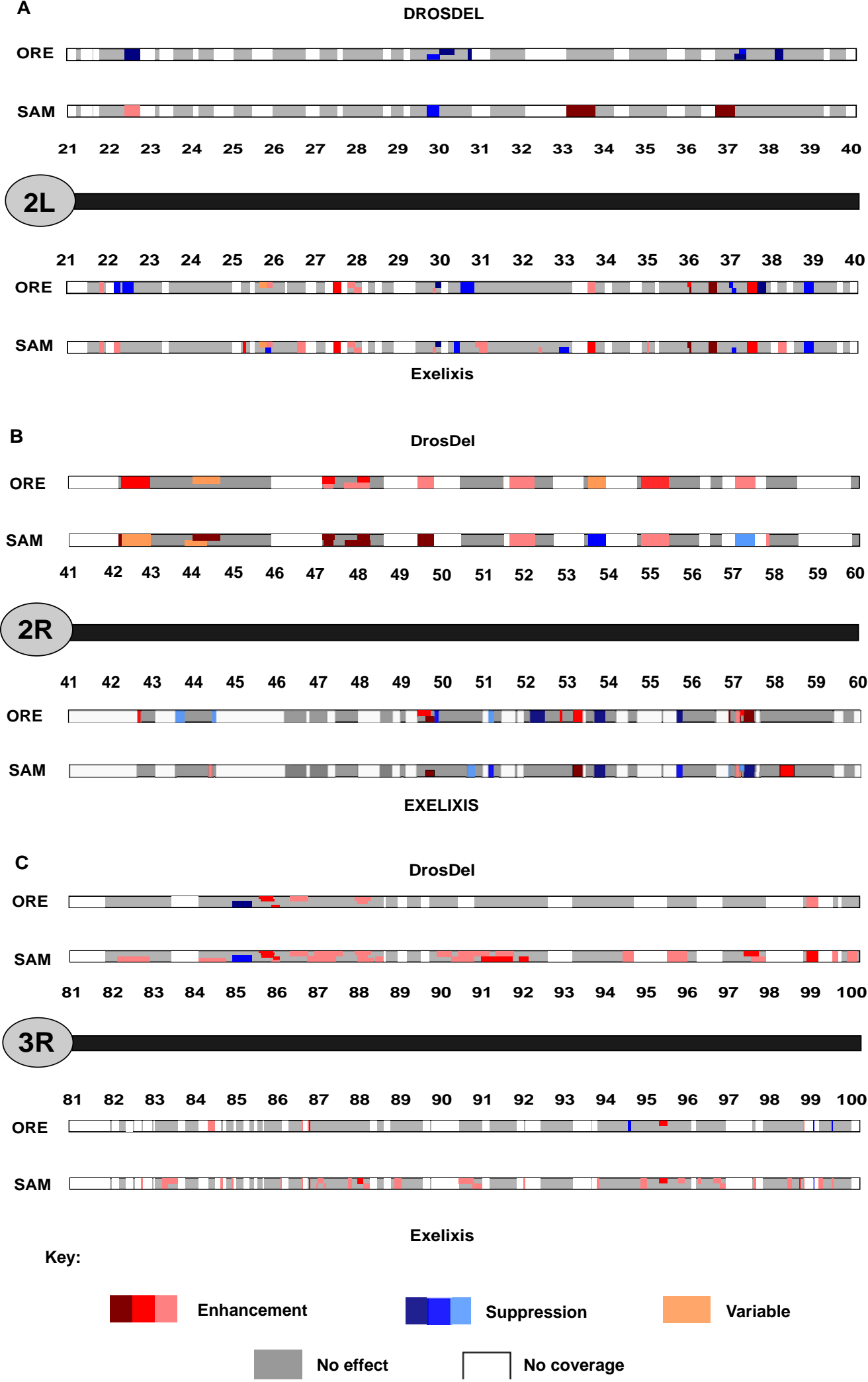

Supplement: Figure S4 — Distribution of modifiers on remaining chromosome arms. Figure legend and description as for figure 3A. A) Chromosome arm 2L. B) Chromosome arm 2R. C) Chromosome arm 3R. (PDF) [file pgen.1003661.s004.pdf]

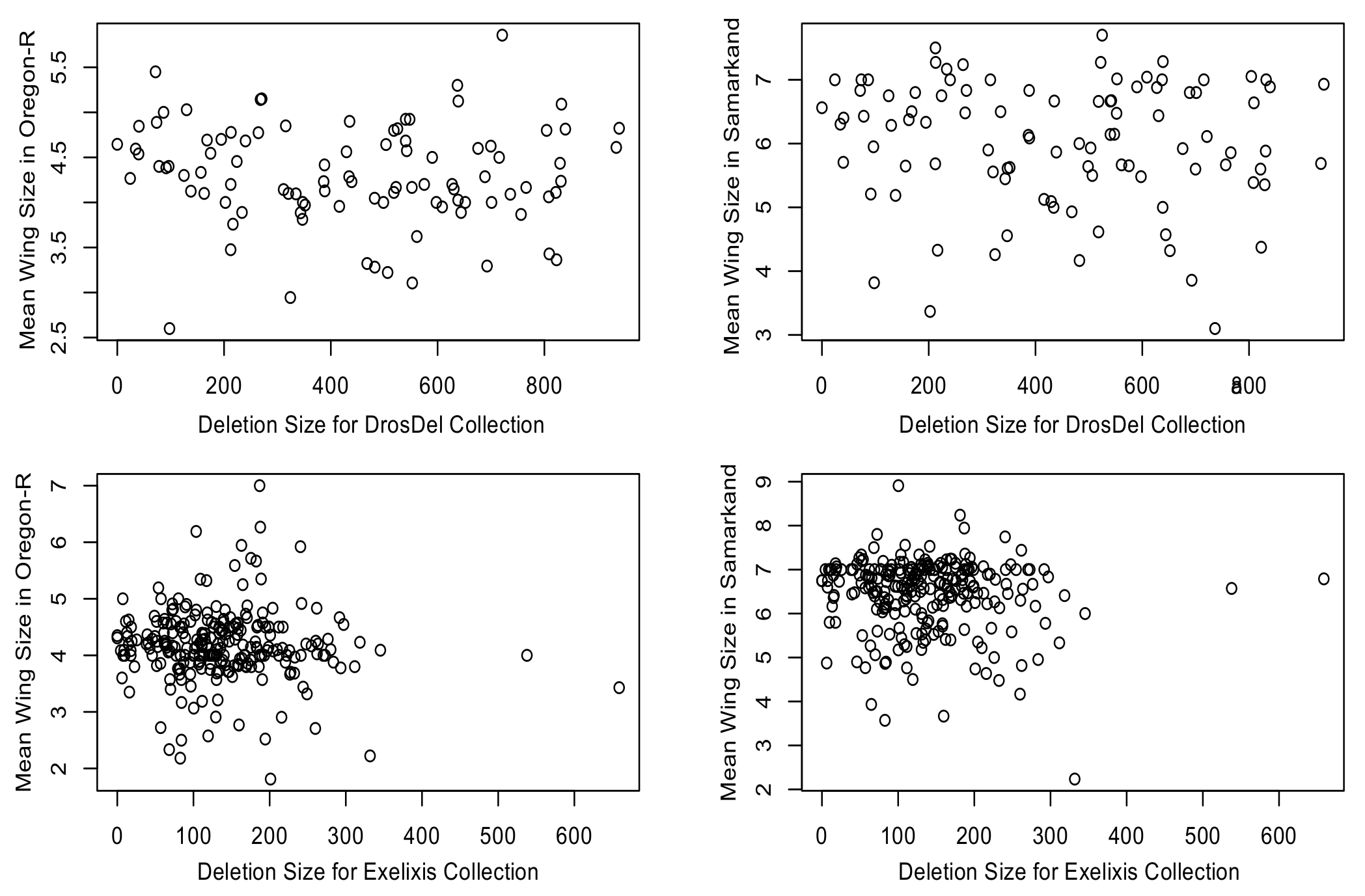

Supplement: Figure S5 — No association between size of the genomic deletion and magnitude of effect as a modifier of sdE3. To determine whether the deletions generally uncovered a single or multiple modifier alleles of sdE3, we examined the relationship between the magnitude of the effect of the deletion on the wing phenotype, and the size of the deletions (in kbp). As seen in these figures, there is no association between them, suggesting that across the set of screened lines, each deletion is likely only uncovering a single modifier allele. However particular individual deletions may have more than one modifier, and modifiers that act in opposite directions. (TIF) [file pgen.1003661.s005.tif]

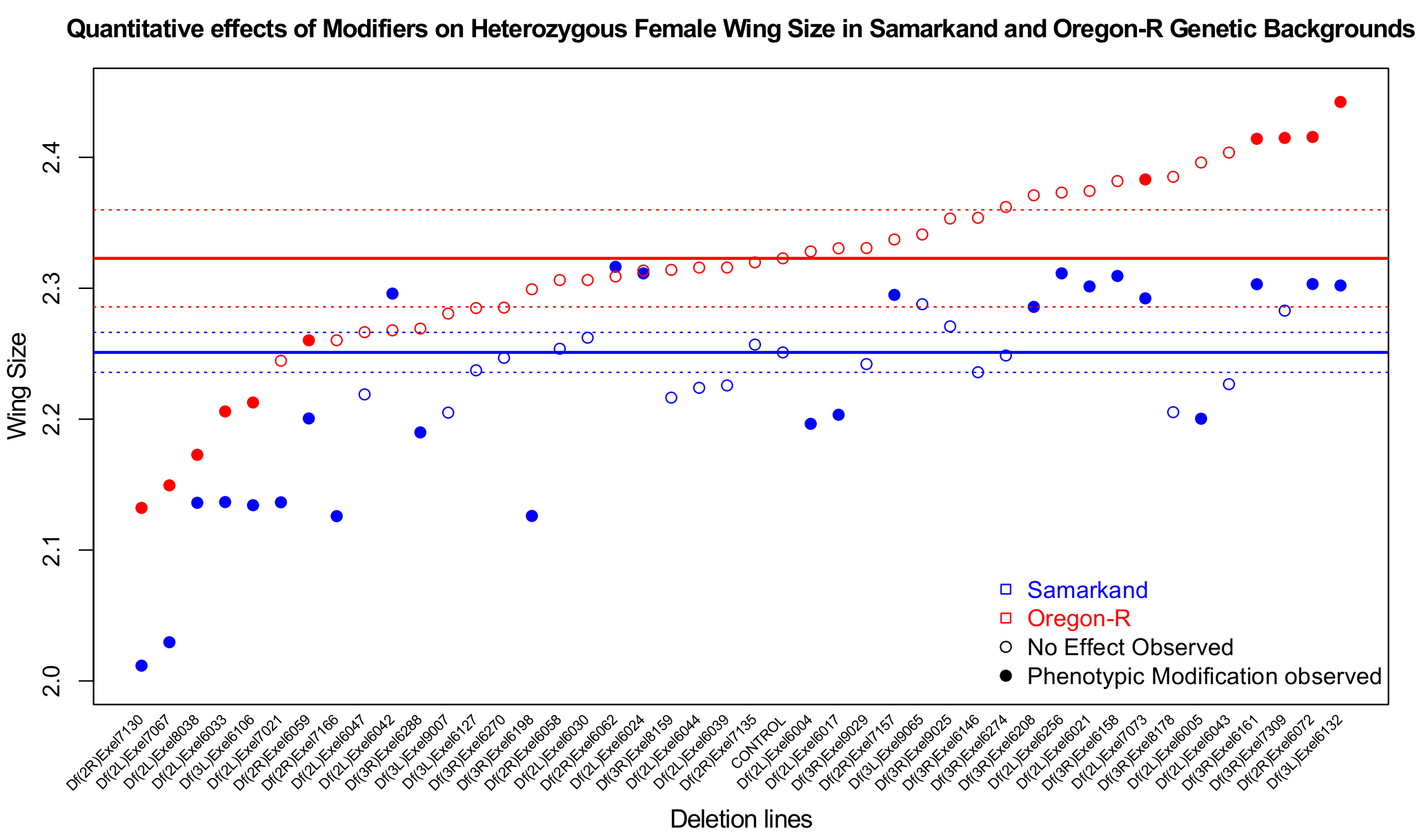

Supplement: Figure S6 — The effects on wing size of 44 deletions in females heterozygous for sdE3. To determine the extent of the phenotypic effects of the genomic deletions on wild-type wing sizes, we examined the effects of 44 of the deletions (the same ones used for Figure 4) in sdE3/+; Deletion/+ females in each background. While the mean wing size differed across wild-type backgrounds, the range of phenotypic effects around each mean was similar (see text). Importantly, the coefficient of variation across strains was ∼10× smaller for wing size for wild-type wings, than for the wings of sdE3 hemizygous males. (TIF) [file pgen.1003661.s006.tif]

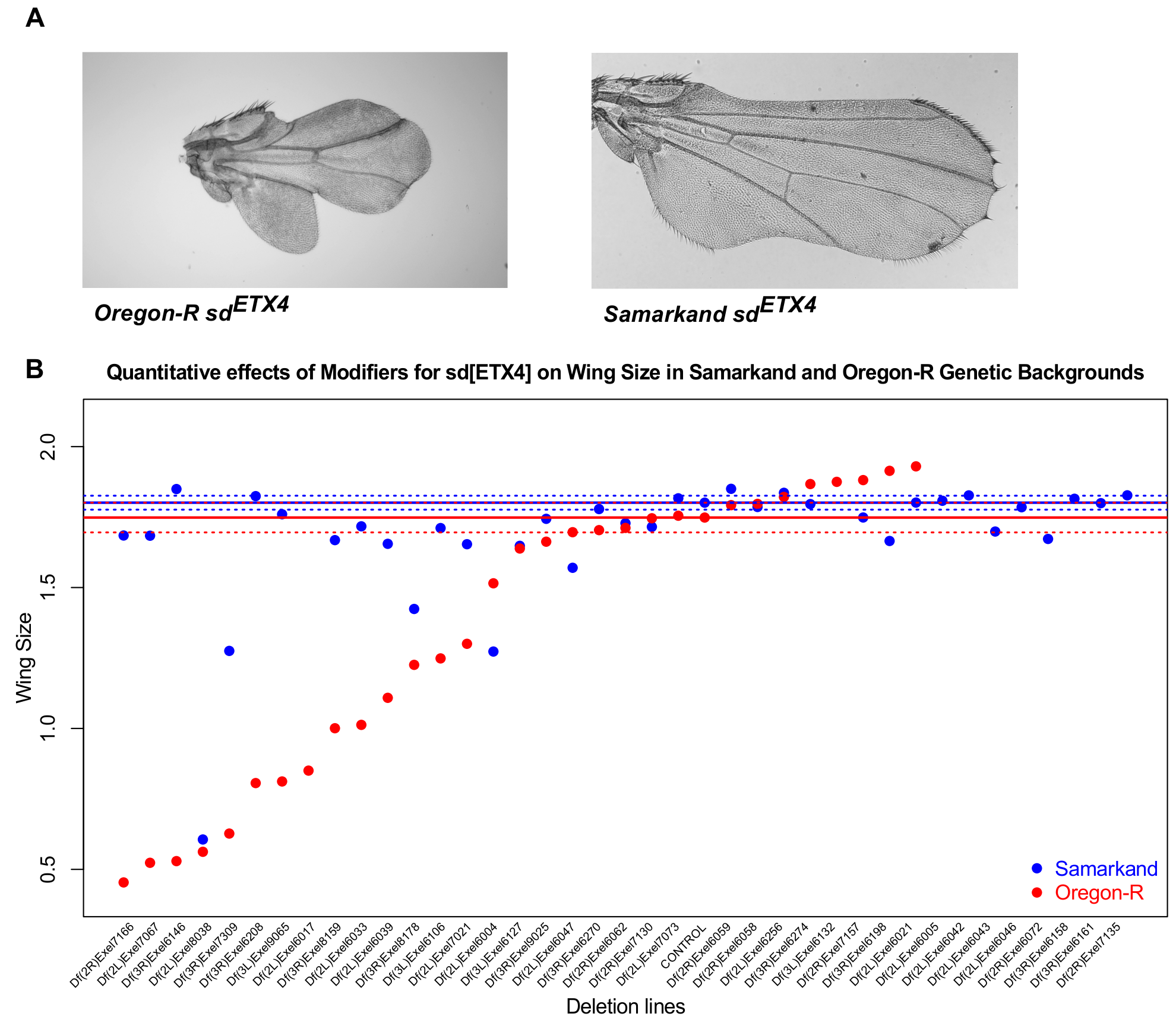

Supplement: Figure S7 — The background dependent effects on the sdETX4 allele. To determine whether the findings observed for the background dependence of the genetic interactions of the sdE3 allele with the deletions would hold across other alleles, we introgressed an additional allele, sdETX4, into both Samarkand and Oregon-R, and re-examined a subset of the deletions. A) sdETX4 also shows profound background dependence with respect to the expressivity of the sd phenotype. As described in the text, the results were significantly correlated across alleles. Interestingly the background dependent expressivity of sdETX4 is substantially weakened in crosses with the Exelixis Deletion progenitor strain. However, the background dependence of the genetic interactions appears to be at least as extreme as that observed for sdE3 (B). (TIF) [file pgen.1003661.s007.tif]
