# Supplementary material for: The Conditional Nature of Genetic Interactions: The Consequences of Wild-Type Backgrounds on Mutational Interactions in a Genome-Wide Modifier Screen
Source: PLoS Genet. 2013 Aug 1;9(8):e1003661. doi: 10.1371/journal.pgen.1003661 (PMC3731224; doi:10.1371/journal.pgen.1003661)
Supplement: Table S2 — Results from fine deletion mapping. (DOCX) [file pgen.1003661.s009.docx]

Supplemental Table 2: Fine mapping of modifiers in segmental deletions.

| Chromosome region | Initial effect | Deletions | Confirmed insertion candidates | confirmed |
| --- | --- | --- | --- | --- |
| 49E1 | Background dependent enhancement (S) | Df(2R)Exel8056  Df(2R)Exel7123  Df(2R)BSC485  Df(2R)ED2308 | PBac{w[+mC]=WH}*vg*^f02736^ | *vg* |
| 57B3-B5 | Background dependent enhancement (O) | Df(2R)Exel6070  Df(2R)Exel7166  Df(2R)BSC702  Df(2R)BSC402  Df(2R)BSC404  Df(2R)BSC814 | No candidate verified |  |
| 63F2-F7 | Background dependent enhancement (S) | Df(3L)Exel6097  Df(3L)Exel6099  *Df(3L)Exel6098*  Df(3L)BSC368  *Df(3L)ED208*  Df(3L)ED4341 | **Enc**, Rdh, Awh  (not arrowhead) |  |
| 86E13-E16 | Background independent enhancement | Df(3R)Exel6161  *Df(3R)Exel7309*  Df(3R)Exel8154  Df(3R)BSC469  Df(3R)ED5516  Df(3R)ED5559 | No candidate verified. |  |
